# Evaluation of MLPA as a comprehensive molecular cytogenetic tool to detect cytogenetic markers of chronic lymphocytic leukemia in Egyptian patients

**DOI:** 10.1186/s43141-021-00198-z

**Published:** 2021-06-28

**Authors:** Ola M. Eid, Rania M. A. Abdel Kader, Lamiaa A. Fathalla, Amany H. Abdelrahman, Ahmed Rabea, Rana Mahrous, Maha M. Eid

**Affiliations:** 1 Human Cytogenetics Department, Human Genetics and Genome Research Division, National Research Centre, Bohouth Street, 12311 Dokki, Cairo, Egypt; 2 Clinical Pathology Department, National Cancer Institute, Cairo University, Cairo, Egypt; 3 Clinical Pathology Department, National Research Centre, Cairo, Egypt; 4 Oncology Department, National Cancer Institute, Cairo University, Cairo, Egypt

**Keywords:** Chronic lymphocytic leukemia (CLL), Chromosomal aberrations, Multiplex ligation-dependent probe amplification (MLPA), Fluorescence in situ hybridization (FISH)

## Abstract

**Background:**

Chronic lymphocytic leukemia (CLL) is the most common form of adult leukemia. This disease is genetically heterogeneous, and approximately 85% of patients with CLL harbor chromosomal aberrations that are considered effective prognostic biomarkers. The most frequent aberrations include deletions in 13q14, followed by trisomy 12, and deletions in 11q22.3 and 17p13 (TP53). Currently, fluorescence in situ hybridization (FISH) is the most widely used molecular cytogenetic technique to detect these aberrations. However, FISH is laborious, time-consuming, expensive, and has a low throughput. In contrast, multiplex ligation-dependent probe amplification (MLPA) is a reliable, cost-effective, and relatively rapid technique that can be used as a first-line screening tool and complement with FISH analysis. This study aimed to evaluate the contributions of MLPA as a routine standalone screening platform for recurrent chromosomal aberrations in CLL in comparison to other procedures. Thirty patients with CLL were screened for the most common genomic aberrations using MLPA with SALSA MLPA probemix P038-B1 CLL and FISH.

**Results:**

In 24 of the 30 cases (80%), the MLPA and FISH results were concordant. Discordant results were attributed to a low percentage of mosaicism. Moreover, the MLPA probemix contains probes that target other genomic areas known to be linked to CLL in addition to those targeting common recurrent CLL aberrations.

**Conclusions:**

The usage of MLPA as the first screening platform followed by FISH technique for only the negative cases is the most appropriate approach for CLL diagnosis and prognosis.

## Background

Chronic lymphocytic leukemia (CLL) is the most common form of adult leukemia in western countries, accounting for 30% of all leukemia cases. However, it is infrequent in the Eastern world. In Upper Egypt, CLL accounts for around 11.3% of all leukemia cases. This hematopoietic neoplasm arises from B-lymphocytes in the peripheral blood, bone marrow, and/or lymph nodes [1, 2]. Moreover, CLL is a genetically heterogeneous disease, and the clinical course may range from months to decades. Approximately 85% of CLL patients harbor chromosomal aberrations, which are considered effective prognostic biomarkers. The most frequent aberrations involve deletions in 13q14 (50–60%), which are associated with a good prognosis. The next most frequent aberration is trisomy 12 (12–25%), which is associated with intermediate prognosis, followed by 11q22.3 (*ATM*; 10–20%) and 17p13 (*TP53*; 5–10%) deletions, which are associated with a poor prognosis. These aberrations are important prognostic biomarkers for treatment decision-making [3].

Currently, fluorescence in situ hybridization (FISH) is the most widespread molecular cytogenetic technique used to detect genetic abnormalities in CLL [4]. However, FISH cannot detect small or intragenic deletions. Moreover, FISH is a laborious, time-consuming, expensive, and low-throughput procedure relative to other molecular genetic procedures used to detect common aberrations. Several other chromosomal aberrations in CLL have been detected using different techniques. However, these aberrations are not usually analyzed in clinical practice [5].

Multiplex ligation-dependent probe amplification (MLPA) was first introduced in 2002 [6]. This multiplex PCR technique can detect abnormal copy numbers in up to 50 different genomic DNA or RNA sequences and can differentiate sequences differing in only one nucleotide. Up to 96 samples can be tested simultaneously by MPLA, and the turn-around time is within 24 h. Consequently, MLPA has considerably increased the detection rates of various genetic disorders [7]. MLPA has also been applied successfully to the detection of copy number abnormalities in various malignant hematopoietic disorders, such as CLL [8]. MLPA is a reliable, cost-effective technique and is more rapid than FISH. Although MLPA cannot detect low-level mosaicism, it remains useful as a first-line screening tool and complement with FISH analysis [9]. The commercially available SALSA MLPA probemix P038 was designed specifically for CLL screening and permits the concurrent evaluation of various risk-linked genomic targets. This kit contains probes for 10q (PTEN), 11q (ATM, RDX, PPP2R1B, CADM1), chromosome 12, 13q14 (RB1, DLEU1/2/7, KCNRG, MIR15A), 14q, 17p (TP53), and chromosome 19.

This study aimed to evaluate the contributions of MLPA as a routine standalone screening platform for recurrent chromosomal aberrations in CLL in comparison to other procedures such as FISH.

## Methods

This study was conducted at the National Research Centre, Egypt, and was approved by its Medical Ethical Committee. Informed written consent was obtained from the study participants. Thirty CLL patients (16 males, 14 females) were included in this study. The average age at the time of sampling was 65 years (range, 36–88 years). All participants attended the National Cancer Institute, Cairo University, Egypt. The diagnosis of CLL was established according to the World Health Organization classification of hematolymphoid tumors [10]. CLL was diagnosed by the presence of at least 5000 monoclonal B-lymphocytes/μl with a CLL immune phenotype in the peripheral blood (PB) for at least 3 months. Typically, CLL lymphocytes are small and mature-looking, with scanty cytoplasm and a dense nucleus containing partially aggregated chromatin. PB samples were collected on heparin to enable blood culture and on K2-EDTA in a vacutainer tube to allow DNA extraction.

### FISH analysis

Peripheral heparinized blood samples were cultured without mitogens and incubated at 37°C for 24 h. Cell harvesting and slide preparation were performed using the standard conventional cytogenetic methods.

FISH analysis was performed according to the manufacturer’s instructions and Pinkel et al. [11], using FISH probes for the most common genomic aberrations associated with CLL, including trisomy 12 and deletions at the 13q14, 11q22, and 17p13 loci. All FISH probes were commercially available (Cytocell, UK). The slides were examined using a suitable filter set on an optimally performing fluorescence microscope with an applied imaging system. A total of 200 interphase cells were examined per patient.

### MLPA assay

DNA was extracted from the PB lymphocytes of all 30 cases and reference samples (one reference sample per seven patient samples, with a minimum of three references per test) using the QIAamp DNA Mini Kit (Germany) according to the manufacturer’s instructions. The quality and quantity of the DNA samples were determined using a NanoDrop spectrophotometer.

The MLPA assay was performed using SALSA MLPA probemix P038-B1 CLL according to the manufacturer’s instructions (MRC-Holland, Netherlands). This probemix comprises multiple probes specific for chromosomal regions and genes associated with recurrent copy number aberrations in B-lymphocyte CLL, including 10q23.31 (*PTEN*), 11q 22 (*ATM*, *RDX*, *PPP2R1B*, *CADM1*), chromosome 12, 13q14 (*RB1*, *DLEU1/2/7*, *KCNRG*, *MIR15A*), 14q, 17p (*TP53*), and chromosome 19. Moreover, the P038 probemix contains three probes to detect the NOTCH1 7541-7542delCT, SF3B1 K700E, and MYD88 L265P mutations, which only produce a signal when the precise mutation is present. The assay kit included SD009 sample DNA as a positive control for the mutation-specific probes and data binning in the fragment analysis.

The DNA denaturation and overnight MLPA probemix hybridization steps were followed by probe ligation and amplification on the following day. The amplified products were separated using an ABI 3500 Genetic Analyzer (Applied Biosystems, USA). The results were interpreted using the Coffalyser.Net software (MRC, Holland). Ratios of <0.75, 0.75–1.30, and >1.3 were considered to indicate deletion, normal, and duplication, respectively.

## Results

Samples from 30 patients with CLL were studied. The FISH and MLPA results are summarized in Table 1.

**Table 1:** Summary of the aberrations detected by MLPA and FISH

	FISH Mosaic (%)	MLPA
1	11q del (53%)	11q del
	Tri 12 (83%)	Tri 12
2^a^	Tri 12 (4%)	-
	13q del (29%)	13q del
3	No Abn	No Abn
4	No Abn	No Abn
5	13q del (30%)	13q del
6	No Abn	No Abn
7	13q del (73%)	13q del
8	No Abn	No Abn
9^a^	Tri 12 (5%)	No Abn
10^a^	Tri 12 (59%)	Tri 12
	13q del (16%)	-
	-	Tri 19
11	13q del (76%)	13q del
12	17p del (87%)	17p del
13	No Abn	No Abn
14	Tri 12 (72%)	Tri 12
	17p del (74%)	17p del
15^a^	Tri 12 (9%)	-
	13q del (79%)	13q del
16	Tri 12 (85%)	Tri 12
17	17p del (80%)	17p del
18^a^	11q del (6%)	-
	Tri 12 (48%)	Tri 12
19	No Abn	No Abn
20	13q del (48%)	13q del
21	No Abn	No Abn
22	11q del (87%)	11q del
	13q del (55%)	13q del
23	No Abn	No Abn
24	13q del (28%)	13q del
25	Tri 12 (27%)	Tri 12
26	Tri 12 (60%)	Tri 12
27	No Abn	No Abn
28	11q del (60%)	11q del
	Tri 12 (50%)	Tri 12
29^a^	Tri 12 (90%)	Tri 12
	-	14q del
30	13q del (68%)	13q del

*No Abn* no abnormalities detected

The discordant results of MLPA and FISH are marked with asterisk ^a^

**Fig. 1:**
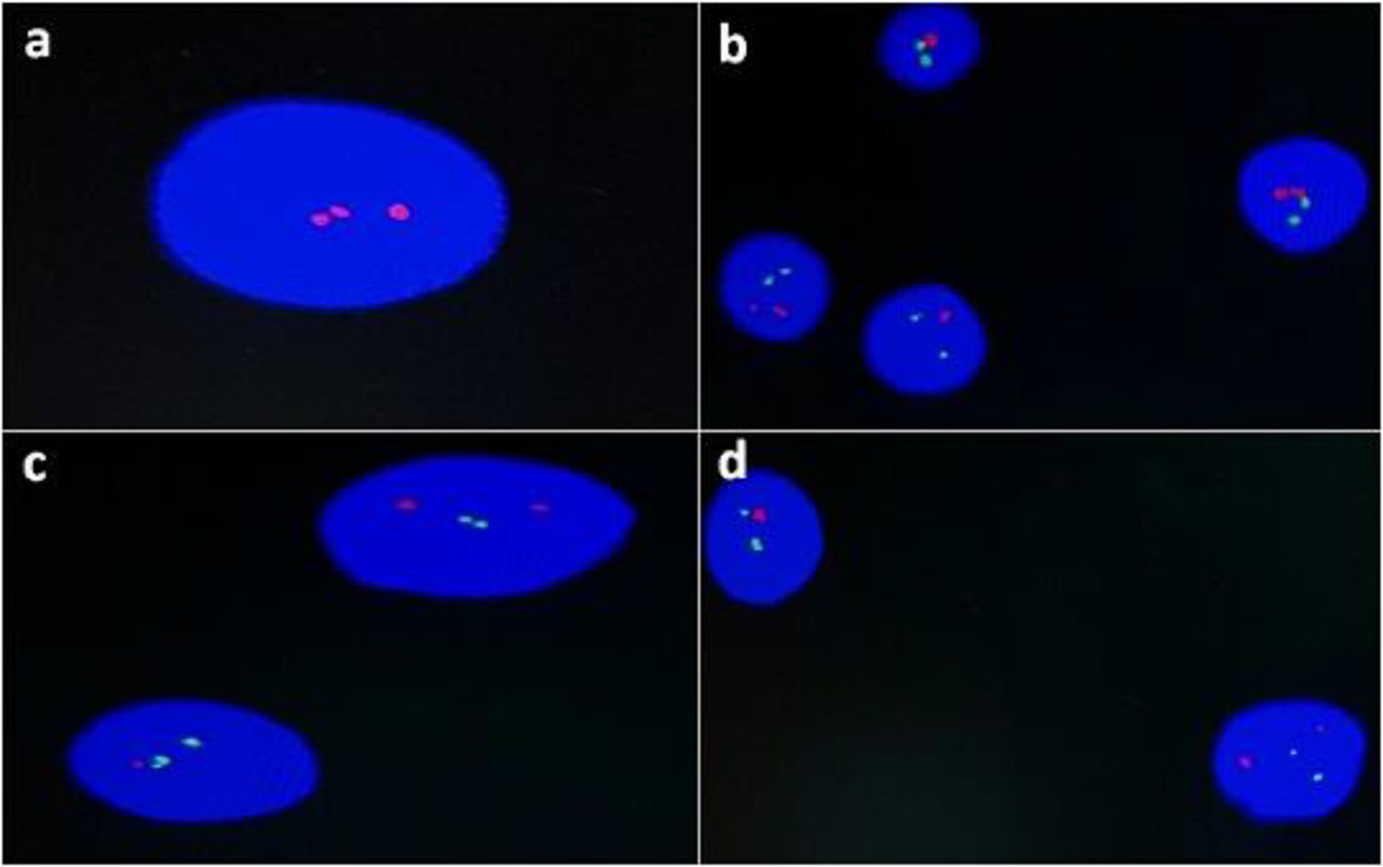
FISH analysis showing **a** trisomy 12 denoted by the presence of three red signals, **b** mosaic positive 11q23 del denoted by the presence of one red signal and two control green signals for centromere 11, **c** mosaic positive 13q14 del denoted by the presence of one red signal and two control green signals for the 13q34 region, and **d** mosaic positive 17p13 del denoted by the presence of one red signal and two control green signals for centromere 17

FISH detected aberrations in 21 cases (70%), whereas no abnormalities were detected in nine cases (30%). The most common defect was trisomy 12, which was present in 12 patients (40%). A 13q14 deletion was detected in 10 cases, while an 11q22 deletion was observed in four cases, and a 17p13 deletion was detected in three cases (Table 2, Fig. 1).

**Table 2:** Frequencies of the abnormalities detected by MLPA and FISH

Abnormality	Genes	Detected by MLPA	Detected by FISH	Result
**11q deletion**	ATM, RDX, PPP2R1B, CADM1	3	4	Disconcordant
**Trisomy 12**	CD27, STAT6, HMGA2, PAH, IGF1	9	12	Disconcordant
**13q14 deletion**	DLEU2, KCNRG, DLEU1, RB1, KCNRG, ATP7B	9	10	Disconcordant
**14q deletion**	AKT1, MTA1, K1AA0125	1	-	Disconcordant
**17p deletion**	TP53	3	3	Concordant
**Trisomy 19**	LDLR, CDKN2D, AKT2, MIR498	1	-	Disconcordant

**Fig. 2:**
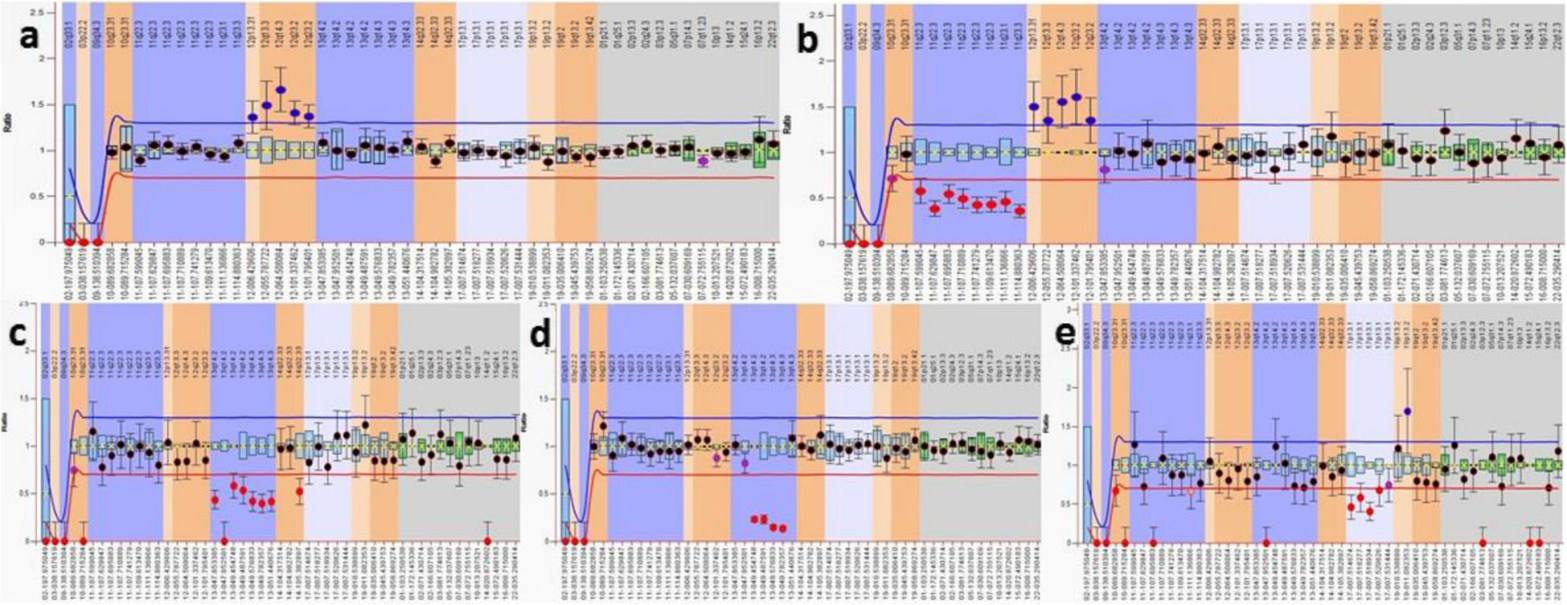
Ratio charts of MLPA results for some CLL patients using SALSA MLPA probemix P038-B1 CLL. Probe ratios below 0.7 (bottom line) or above 1.3 (top line) are usually regarded as indicative of a deletion or duplication, respectively. **a** is a patient having trisomy 12, **b** is a patient having 11q deletion and trisomy 12, **c** is a patient having 13q deletion, **d** is a patient having 13q deletion (this patient is having 30% mosaic 13q deletion by FISH), and **e** is a patient having 17p deletion

MLPA detected aberrations in 20 cases (66.7%) and no abnormalities in the remaining 10 cases (33.3%). The most common abnormality was trisomy 12, which was present in nine cases (30%). A 13q14 deletion was detected in nine cases, while the *RB1* gene was not included in the deleted area in four cases. The 17p13 deletion and 11q22 deletion were detected in three cases each, and the 14q deletion and trisomy 19 were observed in one patient each (Table 2). NOTCH1 7541-7542delCT, SF3B1 K700E, and MYD88 L265P mutations were not detected in any of the patients (Fig. 2).

## Discussion

Many authors have recommended the use of MLPA as an initial diagnostic test [12, 13]. In this study, we aimed to determine the usefulness of the MLPA probemix P038-B1 as a routine standalone screening platform for the detection of clinically relevant chromosome abnormalities in CLL.

Samples from six of the 30 studied patients (20%) yielded discordant MLPA and FISH results. Two cases had a 14q deletion and trisomy 19 respectively, which were not evaluated by FISH in this study. Five cases harbored abnormalities that were identified by FISH but not by MLPA. So, MLPA results were consistent with the FISH results in 24 of 30 patients (80%). Fabris et al. [8] detected a 95% concordance rate between MLPA and FISH results in CLL. The discordant results in these cases may be related to a low level of mosaicism. However, the definition of low mosaicism, or the level at which abnormalities could not be detected by MLPA, has differed between studies and remains controversial. For example, the reported mosaicism thresholds have ranged from 36% in a study by Al Zaabi et al. [9] to 20% in a study by Abdool et al. [14]. However, false-negative MLPA results were reported in samples with an aberrant cell percentage <25% [15, 16]. In our study, abnormalities could be detected by MLPA in a sample containing 27% mosaicism.

Interstitial deletion at 13q14 is the most common chromosomal aberration in CLL and is detected in approximately 50% of cases. The deletion of 13q as the sole abnormality is strongly associated with a favorable disease outcome and a better prognosis. Studies suggest that the clinical course of CLL is accelerated in patients with a large 13q14 deletion that includes the *RB1* gene. Moreover, reciprocal translocations involving 13q14 [t(13q)] and many different chromosomes have been reported. However, the lack of recurrent other abnormalities suggests that the consequence of these translocations is possibly due to the loss of a tumor suppressor gene rather than the generation of a fusion gene [17]. In our study, five of the nine cases in which a 13q deletion was detected by MLPA were affected by large deletions that included the *RB1* gene.

With sufficient accumulated genotoxic damage, CLL cells are directed to undergo cell cycle arrest or apoptosis. *ATM* and *TP53* genes govern the cellular response to DNA damage through the ATM-CHK2-p53 signaling pathway. Alterations of these genes lead to genomic instability and chemoresistance and are associated with adverse prognosis with significantly shorter overall survival [18–20].

11q deletion, which causes a loss of the *ATM* gene at 11q22.3, is detected in 25% of CLL cases. This is the most frequently detected unfavorable genetic anomaly in patients with CLL. Larger 11q deletions also occur and may affect the tumor suppressor genes *PPP2R1B*, *CADM*, and *RDX* [21, 22]. In our study, all patients in whom MLPA detected an 11q deletion were affected by large deletions that included these tumor suppressor genes.

While 17p deletion causes a loss of the tumor suppressor gene *TP53* at 17p13.1 and is associated with a rapid disease progression, poor outcome, drug resistance, and reduced survival duration, in the literature, the incidence of 17p deletion varies widely from 3.4 to 16.8% [23, 24]. In our study, both MLPA and FISH detected 17p deletions in three cases (10%).

Trisomy 12 is the third most common chromosomal aberration detected in patients with CLL. This abnormality is identified in 10–20% of patients [25]. In our study, MLPA and FISH detected trisomy 12 in nine and 12 cases, respectively. This discordance was attributed to the previously discussed low level of mosaicism in three cases.

In addition to four probes that target common recurrent CLL aberrations, the MLPA probemix contains probes that target other genomic areas known to be linked to CLL. These areas, namely, 10q (*PTEN*), 14q, and chromosome 19, are not targeted by the FISH probe panel. *PTEN* is a tumor suppressor gene. *PTEN* is impaired in several types of cancers and plays an important role in CLL pathogenesis. Studies have described defective *PTEN* function in CLL, either through gene mutation/deletion or promoter methylation [26, 27]. While 14q deletions are rare recurrent alterations in CLL frequently associated with trisomy 12, 14q deletions are associated with a short time to treatment. 14q deletions seem to have an adverse prognostic impact when associated with trisomy 12 [28]. Also, trisomy 19 has been detected infrequently in CLL cases and is usually associated with trisomy 12 [29]. In our study, no abnormalities were detected in the 10q (*PTEN*) region. Only one case harbored a 14q deletion, and one case harbored trisomy 19, and both cases were having associated trisomy 12.

Moreover, the P038 probemix includes probes to detect three mutations: NOTCH1 7541-7542delCT, SF3B1 K700E, and MYD88 L265P. These mutations are recently identified as CLL disease parameters. The presence of these mutations is associated with at least one unfavorable prognostic marker [30–33]. However, these mutations were not detected in any of our patients.

## Conclusions

From our study, both assays have comparable capabilities to detect CLL aberrations. MLPA technique is disadvantaged by its inability to detect targeted abnormalities in a sample with a low level of mosaicism. However, the restricted number of regions that can be evaluated by FISH is considered to be disadvantageous. MLPA is more cost-efficient than FISH and encompasses a broader range of target gene loci. Nevertheless, we recommend the usage of MLPA as the first screening platform followed by FISH technique for only the negative cases as the most appropriate approach for CLL diagnosis and prognosis.

## Data Availability

Data and material are available upon request.

## Abbreviations

CLL: Chronic lymphocytic leukemia
DNA: Deoxyribonucleic acid
FISH: Fluorescence in situ hybridization
MLPA: Multiplex ligation-dependent probe amplification
PB: Peripheral blood
PCR: Polymerase chain reaction
RNA: Ribonucleic acid
